# Experimental and numerical evaluation for drum dynamic reliability under extremely complex working conditions

**DOI:** 10.1038/s41598-024-51266-6

**Published:** 2024-01-05

**Authors:** Guochao Zhao, Xin Jin, Lijuan Zhao, Wenchao Zhou, Xuejing Liu

**Affiliations:** 1 School of Mechanical Engineering, Liaoning Technical University, Fuxin, 123000 China; 2 91292 Unit of the People’s Liberation Army of China, Beijing, China

**Keywords:** Engineering, Mechanical engineering

## Abstract

Coal mining machine drums are prone to damage and malfunction under extremely complex working conditions, which seriously affects the efficiency and safety of coal production. In this paper, based on the theory of coal rock cutting and virtual simulation technology, finite element models of drum cutting coal rock were established and then verified by physical experiments. Through simulation analysis, the dynamic reliability of the drum was studied from three aspects: load, stress and wear, and a mathematical model of drum load was established with respect to the traction speed and drum rotation speed; based on the orthogonal test, the optimal working parameters to improve the wear resistance of the drum were derived. The results of the study found that when the traction speed increases, the load on the drum increases, and when the drum rotation speed increases, the load on the drum decreases; when the traction speed is increased from 2 to 6 m/min, the stress on the pick body under different rotation speeds increases to different degrees, with an average increase rate of 27.394%; when the drum rotation speed is 90 r/min, the traction speed is 3 m/min, and the coal loading mode is projectile loading, the wear depth of the picks and spiral blades is relatively small. The research method and results of this paper can provide a reference for the selection of the drum working parameters.

## Introduction

Safety and production efficiency are the main concerns of coal mining enterprises. Yari et al.^1^ proposed an approach to the evaluation and classification of dimensional stone quarries with an emphasis on safety parameters. Subsequently, they investigated a comprehensive model for evaluating occupational and environmental risks of dimensional stone mining^2^. In 2023, Armaghani et al.^3^ proposed and summarized several tree-based solutions for predicting flyrock distance due to mine blasting. In coal mining, the reliability of coal mining machinery has become a focus issue. The drum is the core working mechanism of the coal mining machine, which directly contacts the coal rock material to achieve strong crushing. Wear and failure of drums are inevitable, which is an important factor affecting the dynamic reliability of coal mining machines and curbing the production efficiency of enterprises, and has been widely concerned by scholars. They have carried out relevant research on the load characteristics and wear characteristics of the drum by means of theoretical analysis, experimental research, numerical simulation and so on. Representative achievements in theoretical and experimental research include: Mazurkiewicz^4^ analyzed the influence of the arrangement, size and type of picks on the process of drum cutting coal rock. Jaszczuk^5^ explored the relationship between drum structure, kinematic parameters, body positioning and dynamic loading. Wyk^6^ investigated the cutting process of different shapes of picks at constant speed. In addition, the effects of cutting depth and wear state on the pick load were also found. Wu^7^ used MATLAB optimization toolbox to optimize the structural parameters of the drum with the optimization objectives of cutting specific energy consumption and load fluctuation coefficient. Liu^8^ studied and obtained the mechanical characteristics of drums for cutting coal seams containing gangue. Hao et al.^9^ constructed a coal mining bench suitable for different working modes according to the load distribution law of coal mining machine in the process of coal rock cutting. Li^10^ established a test bench for cutting resistance testing of picks, obtained the resistance variation curve of picks, and analyzed the resistance of picks in forward and oblique feeds. Liu et al.^11^ established an entropy-based combinatorial model through experimental studies, and evaluated the relationship between drum rotation speed and working performance. Hekimoglu et al.^12^ analyzed the effect of spiral angle on drum load, and calculated the optimal spiral angle to minimize the load. Gao et al.^13^ obtained an empirical model for the cutting torque of coal mining machine drums by conducting cutting experiments on drums and picks with different structures. Luo et al.^14^ experimentally studied the load characteristics of coal mining machine drums for three coal seam conditions: coal, rock and mixed coal rock, and analyzed the influence of taper angle, intercept distance, spiral angle, pick arrangement and traction speed on the axial force of the spiral drum. Li et al.^15^ established the mathematical model of cutting thickness and cutting resistance of the pick by judging the cutting conditions in the oblique cutting process. Abu Bakar et al.^16^ compared the axial cutting resistance and radial cutting resistance of picks in cutting dry and wet coal walls through an experimental study. Tiryakil^17^ statistically analyzed the loss frequency of picks on the drum and found that the loss frequency of picks located at the end plates have a higher loss frequency than the picks located at the spiral blades. Gajewski et al.^18^ used discrete wavelet transform for noise reduction of vibration signals during coal rock cutting, and determined the wear degree of the picks by fuzzy neural network, which proved the effectiveness of applying intelligent optimization algorithm to classify the wear degree level the picks. Nahak et al.^19^ investigated the failure phenomenon of WC 94%–CO 6% hard metal alloy tips of radial picks and explored the wear mechanism at the microstructure level. Dewangan et al.^20^ conducted a wear experimental study on mining WC–Co tip of conical pick and explored the wear mechanism of picks from the aspects such as WC particle exfoliation and WC particle porosity. Zhang et al.^21^ conducted a study on pick wear state identification based on wavelet packet and SOM neural networks. Zhang et al.^22^ realized the failure mode recognition of pick alloy head's based on BP neural network. Zhang et al.^23^ studied the coal rock cutting characteristics and peak flash temperature of the picks during the cutting process based on infrared thermal image testing. Eshaghian et al.^24^ analyzed the wear failure form of the spiral drum and optimized the drum. Jakub et al.^25^ established the cutting power and torque data samples of picks under different wear states by testing and constructed a pick wear prediction model using neural network. Macias et al.^26^ investigated the effect of abrasion resistance of soil and rock on the friction loss of mining machinery tools. Hassanpour^27^ established a relationship between tool wear life and effective geological parameters. Comakli^28^ analyzed the impact of geological conditions in Cappadocia, Türkiye, on the wear state of mining machinery tools.

Theoretical and experimental research results have laid the foundation for scholars to analyze the drum load characteristics and reveal the drum wear mechanism, but the experimental research has the defects of high cost, high risk, time-consuming consumables, etc., which impose strict requirements on the research conditions and brings resistance to scholars to carry out the research work. With the rapid development of computer technology, numerical simulation, especially finite element analysis method, plays an important role in the research of drum cutting coal rock. Bo Yu^29^ simulated the dynamic process of coal rock crushing based on finite element method and concluded that shear damage is the main form of coal rock crushing. Brijes Mishra^30^ developed a virtual experimental platform for automated rotary coal mining through finite element numerical simulation, and investigated the effect of size and shape of the pick tip on the cutting performance of the cutting system. MiKl^31^ simulated the cutting process of picks with different geometries using ABAQUS software and analyzed the loads and stresses of the picks. Ji^32^ analyzed the dynamic force of picks under different cutting speeds, cutting thicknesses, cutting cone angles and installation angles by using the finite element method. Wang et al.^33^ analyzed the forces, stresses, and vibration accelerations of the picks using explicit dynamics analysis software LS-DYNA. Moreover, the relationship between the installation angle of the picks and the lumpiness of coal rock was investigated. He et al.^34^ simulated the coal mining process of a thin coal seam shearer drum based on the finite element method and determined the optimal arrangement of the picks on the drum. Guo^35^ analyzed the dynamic response of the cutting system of coal mining machine in cutting coal seam by using explicit dynamics analysis software LS-DYNA, and investigated the effects of different drum rotation speeds, traction speeds, and hydraulic stiffness of lifting cylinders on the dynamic characteristics of the cutting system. In addition, the load information of each pick during the drum cutting coal rock was obtained. Wan et al.^36^ analyzed the dynamic response and reliability of shearer drum in coal mining process by using the finite element method. Somanchi et al.^37^ analyzed the effect of design variables on the drum performance, and used virtual prototyping technology to realize the parametric design of the drum and the calculation of the pick force. Liu et al.^38^ simulated the process of pick cutting coal rock based on finite element method, studied the effect of cutting mode and inclination angle on coal rock stress, and explored the wear mechanism of pick based on the rock stress state. Liu^39^ established the contact model between the picks and coal rock materials based on theoretical analysis and finite element simulation, and analyzed the wear characteristics of the picks.

The above research results fully demonstrate the feasibility of utilizing the finite element method to study the drum cutting coal rock process. However, with the development trend of coal mining, most coal miners are now working in thin coal seams with relatively poor conditions, such as gangue, sulfide iron nodules and other harsh working conditions. The reliability and friction loss of the drums are especially prominent, and have become a key issues to be urgently solved. However, most scholars have not yet fully considered the complex coal seam conditions such as gangue in their research process, and the research on the drum wear has rarely been reported. Therefore, on the basis of existing research, this paper adopts the finite element method to establish the dynamic coupling model of coal rock cutting, and conducts research on the dynamic reliability of the drum from three aspects of load, stress and wear. The research results can provide reference for improving the reliability and performance of the drum.

The organizational structure of this article is as follows: the constitutive model of coal rock materials, the dynamic model of pick, and the mechanical model of spiral blade are given in Section “Theoretical model”. The finite element model construction process, including 3D model construction, parameter settings and constraint addition, is presented in Section “Finite element model”. The representative numerical simulation results and physical experiments are discussed in Section “Verification”, verifying the accuracy of the numerical model. The dynamic characteristics of drum during cutting coal rock are analyzed in Section “Dynamic reliability analysis of drum”, including load characteristics and wear characteristics. Section “Conclusion” presents the conclusions of this paper.

## Theoretical model

In the process of coal mining, the drum undertakes the two tasks of cutting and loading. The picks on the drum are in direct contact with the coal rock wall and crush it. Except for the broken coal rock thrown down directly, the broken coal rock in the storage space of the drum are conveyed to the scraper conveyor by the spiral blades on the drum. Therefore, in the entire cutting and loading process of the drum, the picks and spiral blades are the key components, and there are complex interactions with the coal rock materials.

### Pick dynamics model

**Figure 1:**
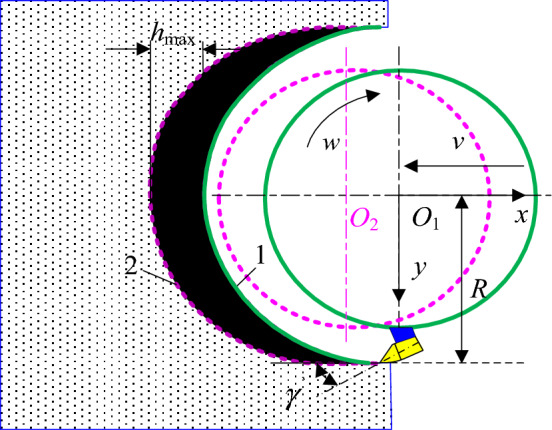
Cutting trajectory lines of the pick.

During the cutting process, the drum rotates axially and pulls forward. The cutting trajectory lines of the pick on the drum (noted as No. 1 and No. 2) are shown in Fig. 1, which can be expressed in turn as

1 $$\left\{ {\begin{array}{*{20}l} {x = - \left( {\frac{50vt}{3} + R\sin wt} \right)} \hfill & {\quad t \in \left( {aT\sim aT + \frac{T}{2}} \right)} \hfill \\ {y = R\cos wt} \hfill & {\quad a = 0,1, \ldots ,T = \frac{2\pi }{w}} \hfill \\ \end{array} } \right.$$

2 $$\left\{ {\begin{array}{*{20}c} {x^{\prime}= - \left( {\frac{{h_{\max } }}{m} + \frac{50vt}{3} + R\sin wt} \right)} \\ {y^{\prime}= R\cos wt\begin{array}{*{20}c} {\begin{array}{*{20}c} {\begin{array}{*{20}c} {} & {} \\ \end{array} } & {} & {} \\ \end{array} } & {} & {} \\ \end{array} } \\ \end{array} } \right.$$

Therefore, the cutting thickness can be described

3 $$h = \sqrt {x^{\prime 2} + y^{\prime 2} } - \sqrt {x^{2} - y^{2} } = \frac{10}{{3mw}}\sqrt {\begin{array}{*{20}l} {w^{2} m^{2} \left( {25v^{2} t^{2} + 0.09R^{2} + 3vrR\sin wt} \right)} \hfill \\ { + 20\pi mw\left( {5v^{2} t + 3Rv\sin wt} \right) + + 100\pi^{2} v^{2} } \hfill \\ \end{array} } - \frac{50}{3}\sqrt {v^{2} t^{2} + \frac{3}{25}vtR\sin wt + \frac{9}{2500}R^{2} }$$

where *R* is cutting radius, mm; *w* is the rotational angular speed of the drum, rad/s; *v* is the traction speed, m/min; *t* is cutting time, s; maximum cutting thickness $h_{{{\text{max}}}} = 100\pi v/3w$. *m* is the number of picks on the same cutting line.

Moreover, the fore of pick is calculated differently when the material of the cutting object is different. When the cutting material is pure coal, the cutting resistance *Z*_0_ and traction resistance *Y*_0_ are calculated according to Eqs. (4) and (5). While, when the cutting material is rock, the cutting resistance *Z*_*j*_ and traction resistance *Y*_*j*_ are calculated according to Eqs. (6) and (7).

4 $$Z_{0} = 10\overline{{A_{p} }} \frac{{0.35b_{p} + 0.3}}{{b_{p} + K_{\psi } \left( {h_{\max } \sin \theta } \right)^{0.5} }}h_{\max } \sin \theta \cdot t_{cp} K_{z} K_{y} K_{\phi } K_{C} K_{ot} \frac{1}{\cos \beta ^{\prime}}$$

5 $$Y_{0} = \left( {0.5 \sim 0.8} \right)Z_{0}$$

6 $$Z_{j} = P_{K} \left[ {k_{T} k_{\psi } k^{\prime}_{\psi } k_{d} k^{\prime}_{y} \left( {0.25 + 1.8h_{\max } \sin \theta \cdot t_{cp} } \right) + 0.1S_{j} } \right]$$

7 $$Y_{j} = \frac{{0.25Z_{j} \left( {0.15 + 0.00056P_{K} } \right)}}{{\left( {10h_{\max } \sin \theta } \right)^{0.4} }}$$

where $\overline{{A_{p} }}$ is the average value of cutting impedance of coal seam in the non-ground pressure affected area, N/mm; $b_{p}$ is the calculated width for the working part of the pick, cm; $t_{cp}$ is the cutting width of pick, cm; $K_{z}$ is the exposed free surface coefficient. $K_{y}$ is the influence coefficient of cutting angle. $K_{\phi }$ is the influence coefficient of the front edge shape of pick. $K_{C}$ is the influence coefficient of pick arrangement. $K_{ot}$ is the influence coefficient of ground pressure on coal wall. $\beta{\prime}$ is the deflection angle of pick, °; $K_{\psi }$ is the brittleness coefficient of coal. $\theta$ is the position angle of pick, °; $P_{K}$ is the contact strength of rock, MPa; $k_{T}$ is the type coefficient of pick. $k_{\psi }$ is the shape coefficient of cemented carbide head. $k^{\prime}_{\psi }$ is the shape coefficient of pick head. $k_{d}$ is the diameter coefficient of cemented carbide head. $k^{\prime}_{y}$ is the influence coefficient of cutting angle of pick. $S_{j}$ is the projected area of wear surface of blunt pick on cutting plane, cm^2^.

The lateral resistance *X*_*j*_ is a function of the cutting resistance, according to which

8 $$X_{j} = Z_{j} \left( {\frac{{C_{1} }}{{C_{2} + 10h_{\max } \sin \theta }} + C_{3} } \right)\frac{{h_{\max } \sin \theta }}{{t_{cp} }}$$

where the determination of *C*_1_, *C*_2_ and *C*_3_ is related to the pick arrangement.

### Spiral blade mechanical model

**Figure 2:**
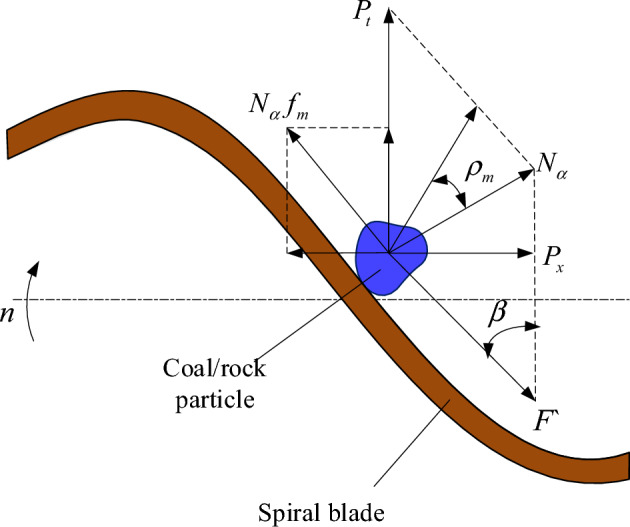
Mechanical model between the single particle and spiral blade.

Because the mass of coal rock blocks is much smaller than that of spiral blades, the effect of the gravitational force of coal rock blocks can be ignored for the purpose of describing the interaction between spiral blades and crushed coal rock. Therefore, according to the theory of granular mechanics, the mechanical model between a single coal rock particle and the spiral blade is shown in Fig. 2.

The coal loading power is given according to

9 $$N_{Z} = 0.001N_{\alpha } v_{t} \left( {\sin \beta + f_{m} \cos \beta } \right)$$

where $N_{\alpha }$ is the normal pressure between spiral blade and particle, N; $v_{t}$ is the tangential parting speed of particle, m/min; $\beta$ is the spiral angle, °; $f_{m}$ is the friction coefficient between particle and spiral blade.

Additionally, the coal loading power can also be expressed as

10 $$N_{Z} = \frac{{vv_{j} K_{J} }}{600n}$$

where $v_{j}$ is the cutting speed of pick tip, m/s; $K_{J}$ is the coal loading resistance coefficient. *n* is the rotation speed, r/min;

Substituting Eq. (10) into Eq. (9) will obtain

11 $$N_{\alpha } = \frac{{5\cos \rho_{{mvv_{j} K_{J} }} }}{{3\pi n^{2} D\sin \beta \sin \left( {\beta + \rho_{m} } \right)\left( {\sin \beta + f_{m} \cos \beta } \right)}}$$

where $\rho_{m}$ is the friction angle. *D* is the drum diameter.

## Finite element model

**Figure 3:**
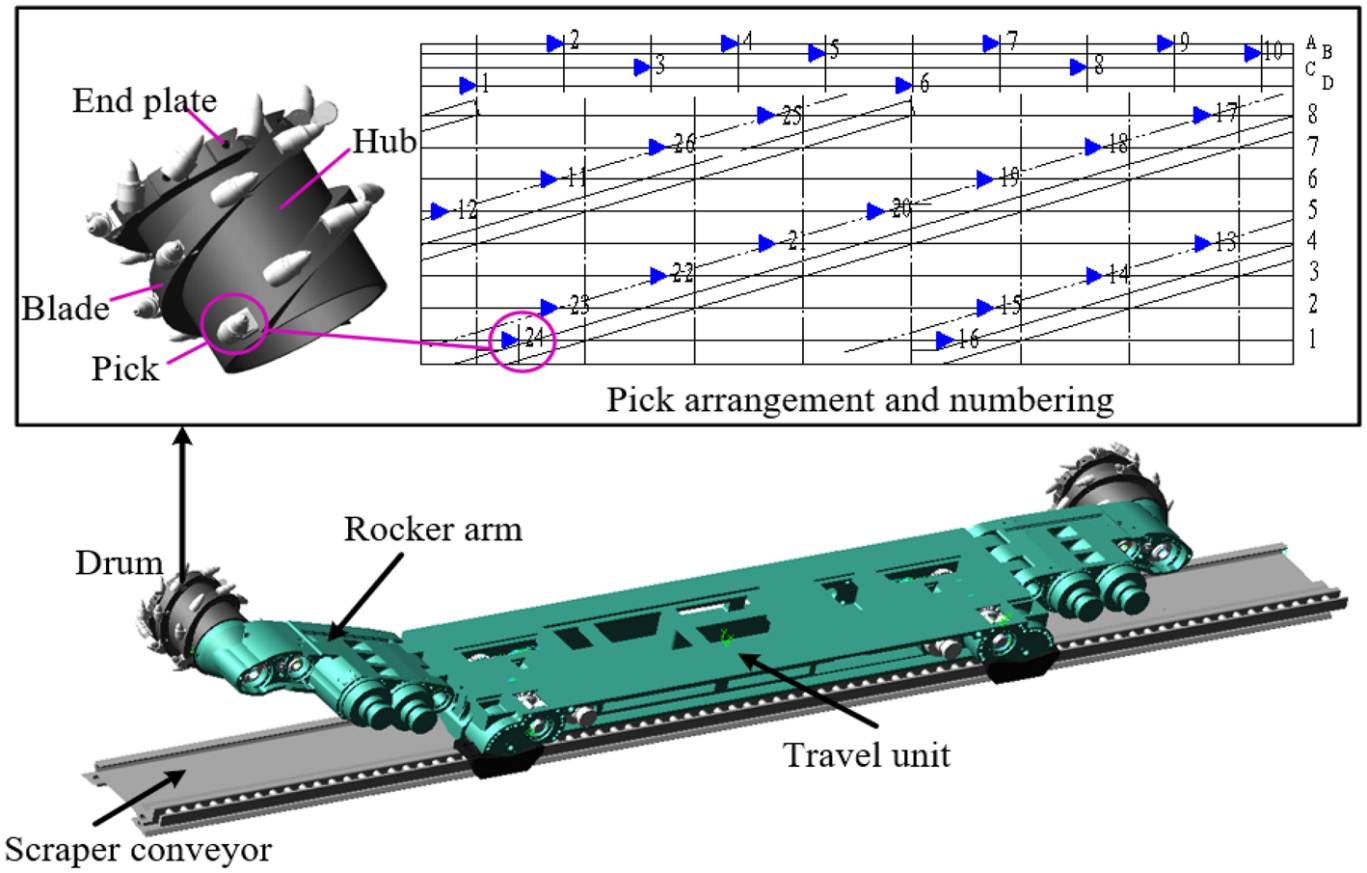
Shearer drum model.

**Figure 4:**
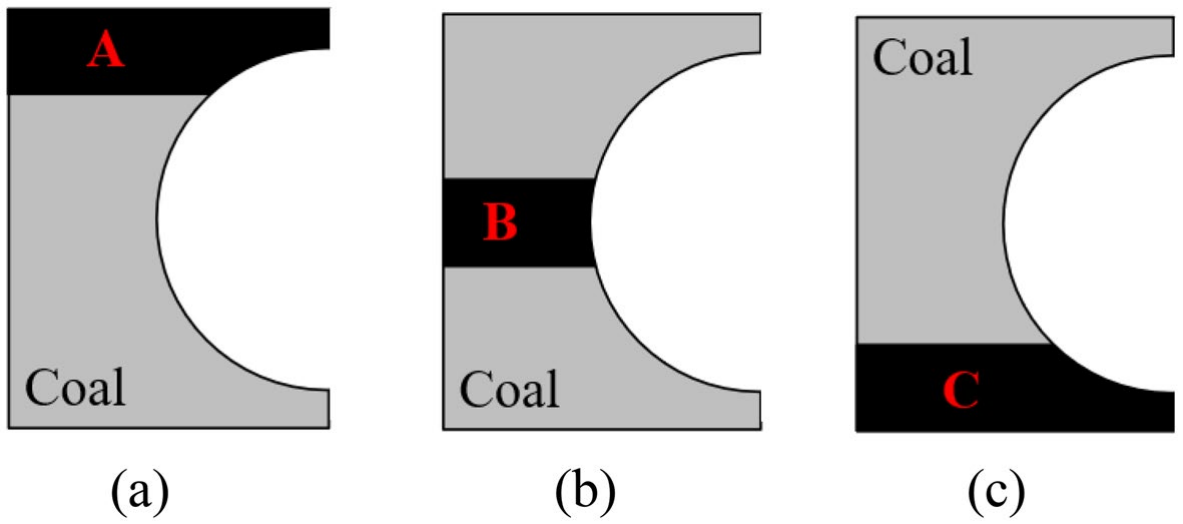
Rock distribution. (**a**) Top layer; (**b**) Middle layer; (**c**) Bottom layer.

In this study, the finite element software LS-DYNA was used for model construction and simulation, which has a lot of geotechnical material constitutive models and unit extinction function, and can be used to simulate the crushing process of coal walls. Material parameters, contact parameters and motion parameters, etc. need to be set during the simulation and analysis. Generally, the finite element simulation calculation often takes a lot of time. Therefore, in order to simulate the process of drum cutting coal rock materials and to reduce the solution time, it is assumed that the coal wall model has been cut into the same free surface as the outer envelope of the drum. In this study, the MG2 × 70/325-BWD shearer drum is the research object (Fig. 3), and the 17th layer of Yangcun Coal Mine in Yanzhou Mining Area is the engineering background, which has complex geological conditions and is often distributed with rock layer. The overall distribution of rock layers can be roughly divided into three types: top layer, middle layer, and bottom layer, as shown in Fig. 4. According to the actual position of the rock layer, three typical coal wall models are established respectively, assembled with the drum model, and then imported into the finite element software for subsequent processing, which are used to simulate the working conditions of the drum cutting complex coal seams.

**Figure 5:**
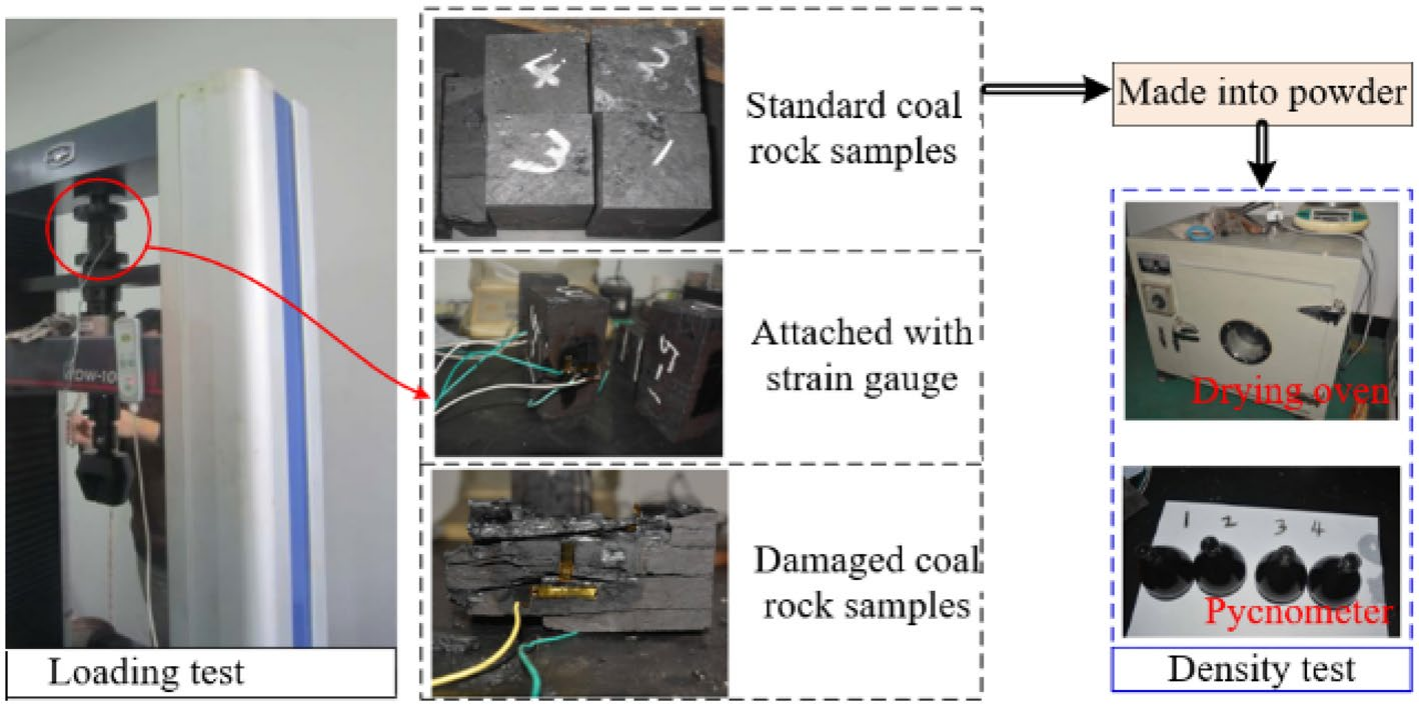
Coal rock property testing.

The 17th coal seam of Yangcun Coal Mine in Yanzhou Mining Area is a complex and thin coal seam, containing sulfide iron nodules and gangue. The coal seam is mainly composed of bright coal with a strip like structure, belonging to high volatile matter, extra-low phosphorus, high sulfur, medium to strong cementation, low to medium ash coals, containing SiO_2_, Al_2_O_3_, Fe_2_S_3_, etc., with a sulfur content of up to 45%. and sulfur in the form of Fe_2_S_3_ accounts for approximately 62% of the total sulfur. The lithology is mainly gray siltstone and dark gray mudstone, interbedded with gray-gray green medium sandstone, gray aluminum mudstone, and limestone. The distribution of rock layers can be classified into three types: A, B, C, as shown in Fig. 4, among which, the top plate (Fig. 4a) is mostly limestone with high hardness, and the local phase changes to siltstone, sandy mudstone or marl, and the bottom plate (Fig. 4c) is aluminum mudstone which is easily muddied in contact with water. It is a typical thin coal seam with complex occurrence conditions. To carry out the coal rock material property test, coal rock samples selected from 17th layer of Yangcun Coal Mine in Yanzhou Mining Area were cut into standard samples of 30 cm × 30 cm × 30 cm, as shown in Fig. 5. In the uniaxial compression test, a strain gauge was pasted in the middle of the sample to be tested and placed on the universal testing machine, and loaded at a speed of 10–50 mm/min until it was destroyed; in the density test, the standard sample was made into powder, and the density of the coal and rock was measured by using the pycnometers and drying oven. The property parameters of coal rock materials were statistically obtained as shown in Table 1. The material parameters of MG2 × 70/325-BWD shearer drum are shown in Table 2. In addition, an 8-node solid164 unit was selected to mesh the drum and coal wall, and a file in .K format was generated.

**Table 1:** Material property parameters of coal rock.

Material properties	Coal	Rock
Density (kg/m^3^)	1.32 × 10^3^	2.40 × 10^3^
Elastic modulus (MPa)	4112	7670
Poisson's ratio	0.23	0.2
Cohesive force (MPa)	1.45	11.5
Internal friction angle (°)	58	38
Compressive strength (MPa)	5.23	52

**Table 2:** Material parameters of drum.

Material properties	Cemented carbide head	Pick body	Pick seat, Hub, blade, End plate
Density (kg/m^3^)	1.46 × 10^4^	7.85 × 10^3^	7.85 × 10^3^
Elastic modulus (MPa)	5.9 × 10^5^	2.07 × 10^5^	2.187 × 10^5^
Poisson's ratio	0.23	0.256	0.3
Yield strength (MPa)	3350	1210	766.67
Tensile strength (MPa)	3500	1403	862.03
Allowable stress (MPa)	1340	754.7	463

**Figure 6:**
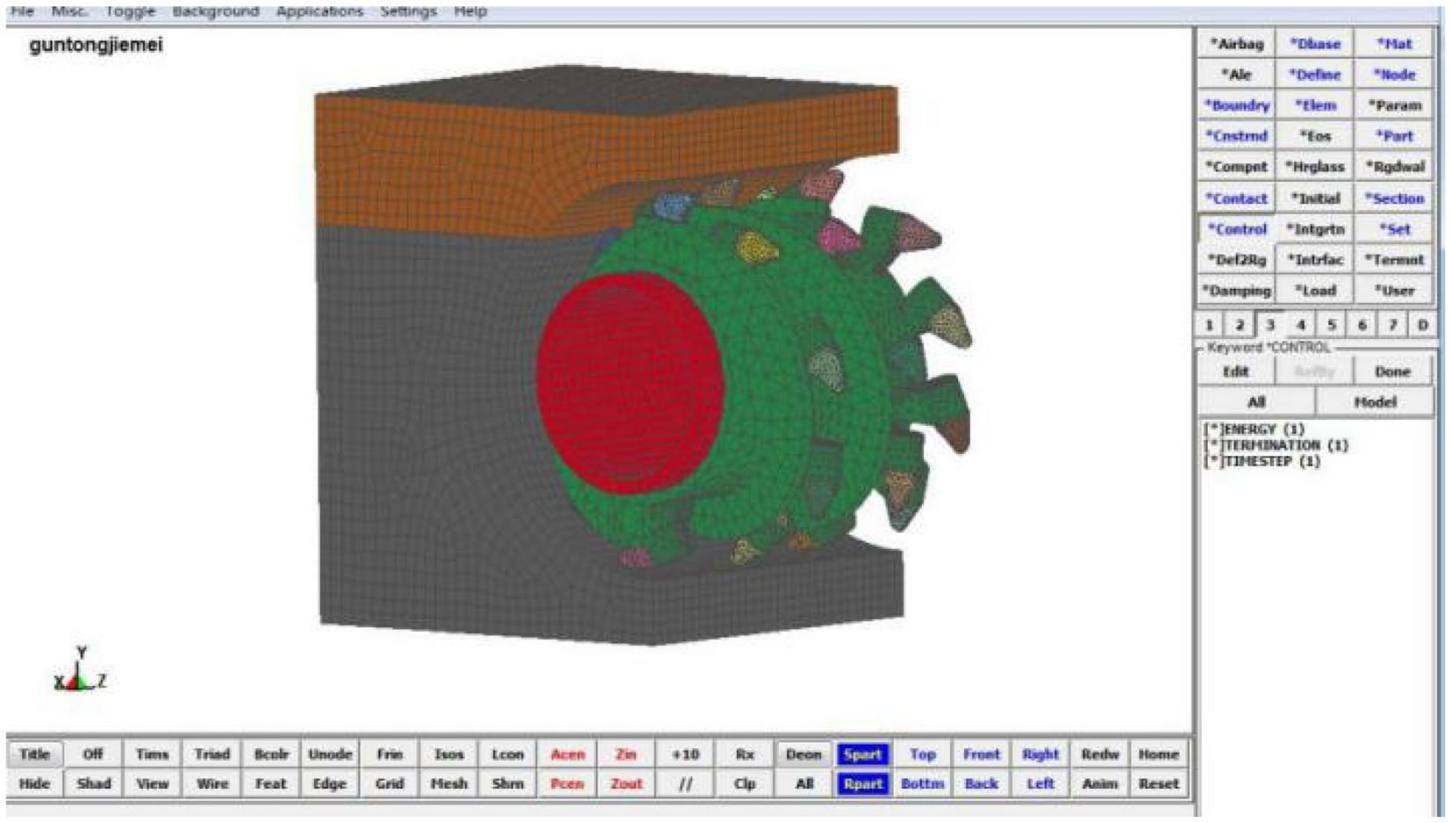
Finite element model of the drum cutting coal wall.

Open the .K file to further define the contact behavior between drum and coal wall. To ensure that the picks are always in contact with the coal rock materials during the cutting process, the contact type between drum and coal wall is selected as face-to-face erosion contact, and the picks are set to be the primary contact and the coal wall is the secondary contact. In addition, the boundary constraints are imposed to the coal wall by the keyword *BOUNDARY_SPC_SET to ensure that the coal wall is not displaced during the cutting process. The reflection-free boundary condition is imposed on the coal rock by the keyword * BOUNDARY_NON_REFLECTING to eliminate the influence of reflection and shear waves on the coal rock unit to be broken. The connection between the square head and the drum is defined by the keyword *CONSTRAINED_EXTRA_NODES_SET. The connection between the pick handle and the pick seat is defined by the keyword *EXTRA-NODES. Through the keyword *MAT_ADD_EROSION, the failure stress of coal material is defined as 5.23 MPa, the failure stress of rock material is defined as 52 MPa. The solidification failure at the coal rock interface is defined by the keyword *CNSTRND_TIEBREAK. In order to obtain drum wear information, it is necessary to define the failure stress of the cemented carbide head as 3500 MPa, the failure stress of the pick body as 1403 MPa and the failure stress of the spiral blade as 862.03 MPa. The rotation speed and traction speed of the drum are defined by the keyword *DEFINE_CURVE. The simulation time is defined as 2 s by the keyword *CONTROL_ TERMI-NATION, and the hourglass energy is set as 10% of the total energy. Finally, the finite element models of the drum cutting coal wall are established, in which the rock layer is located at position A as an example, as shown in Fig. 6.

## Verification

**Figure 7:**
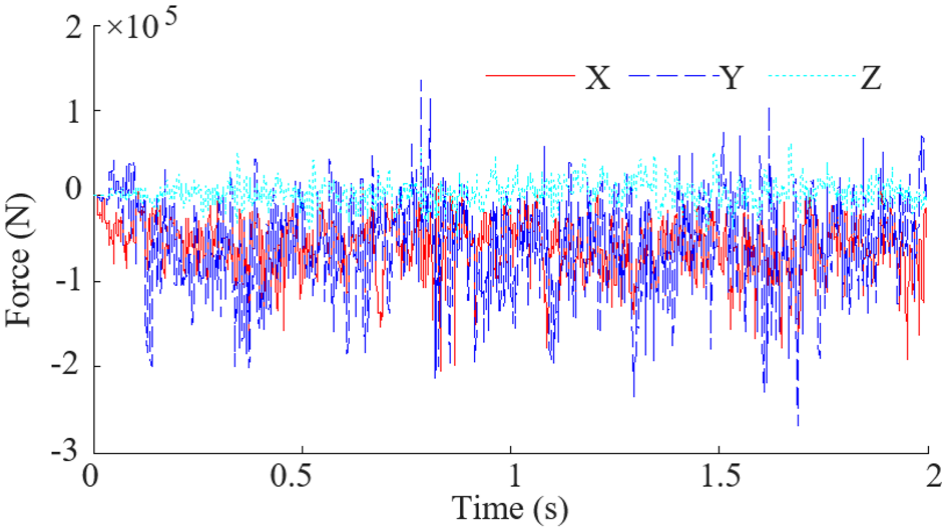
Force curves of the drum.

**Figure 8:**
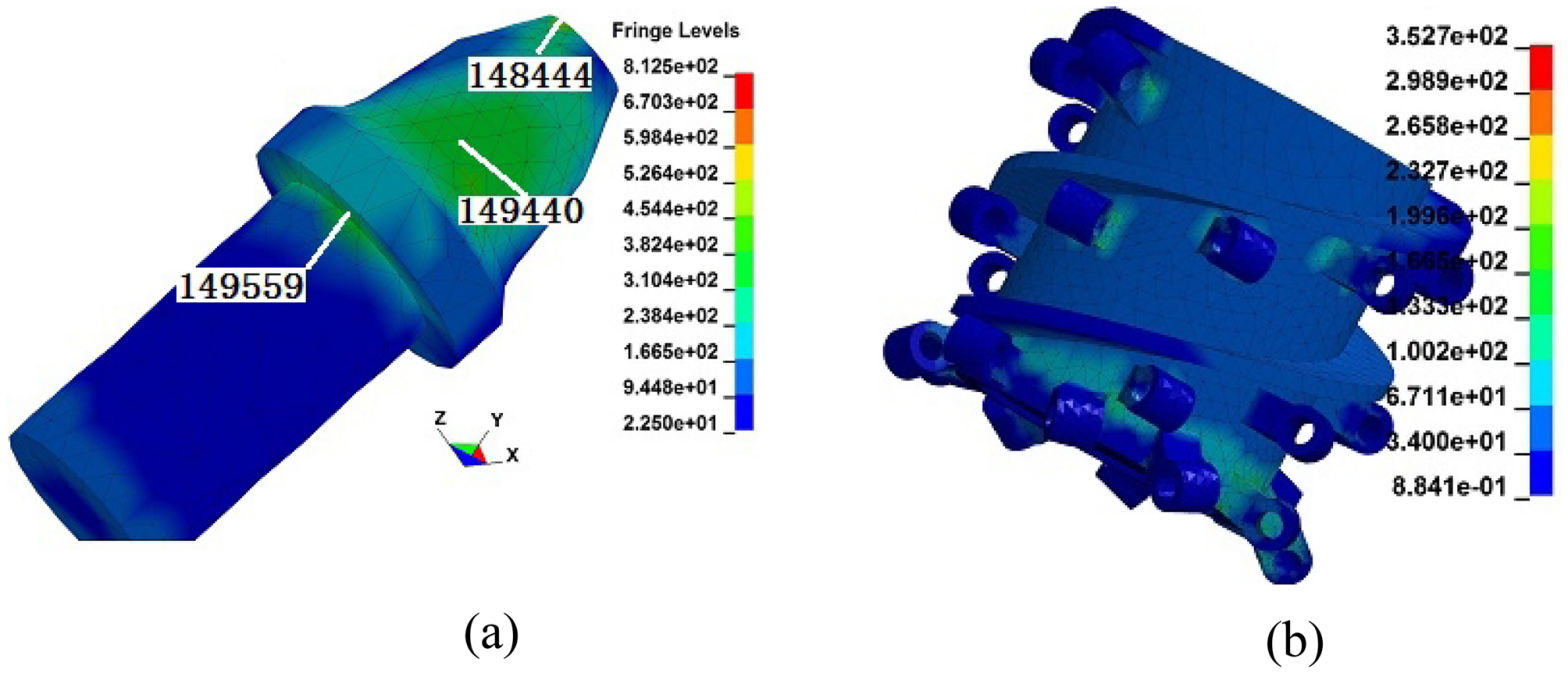
Stress cloud diagrams of the drum. (**a**) Pick body; (**b**) pick seat and spiral blade.

Based on the actual working conditions of the coal mining machine underground, a simulation of drum cutting the coal wall with the rock layer located at position A is performed at a traction speed of 4 m/min and a rotation speed of 80 r/min. The force curves and stress cloud diagrams of the drum were obtained, as shown in Figs. 7 and 8.

As shown in Fig. 7, X, Y and Z represents the force on the drum in the cutting direction, the traction direction and the lateral direction respectively. There is a significant impact force during the process of drum cutting and transporting coal and rock. Among them, the Y-direction force fluctuates most acutely, while the Z-direction force fluctuates relatively gently. According to statistics. The peak value of the X-direction force is 2.05 × 10^5^ N, and the average value is 3.21 × 10^4^ N. The peak value of the Y-direction force is 2.67 × 10^5^ N, and the average value is 4.01 × 10^4^ N. The peak value of the Z-direction force is 6.56 × 10^4^ N, and the average value is 7.53 × 10^3^ N.

As shown in Fig. 8, there is varying degrees of stress concentration in various parts of the drum during the cutting process. The stress values of the 148,444 unit at the top of the pick body, the 149,559 unit at the shoulder of the pick handle head, and the 149,440 unit on the front edge of the pick body cone section are relatively high. The stress of the pick seat is mainly concentrated at the root, and the stress on the pick seat on the end plate is significantly greater than that on the pick seat on the blade. The stress on the blade is mainly concentrated at the outer edge and the welding area with the pick seat. Under this working condition, although the stress in each weak part of the drum does not exceed the yield strength of the material, it is highly susceptible to failure forms such as wear, damage and fracture under long-term strong impact loads.

**Figure 9:**
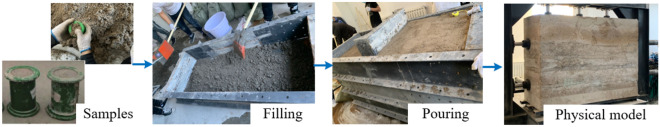
Coal wall physical model.

**Figure 10:**
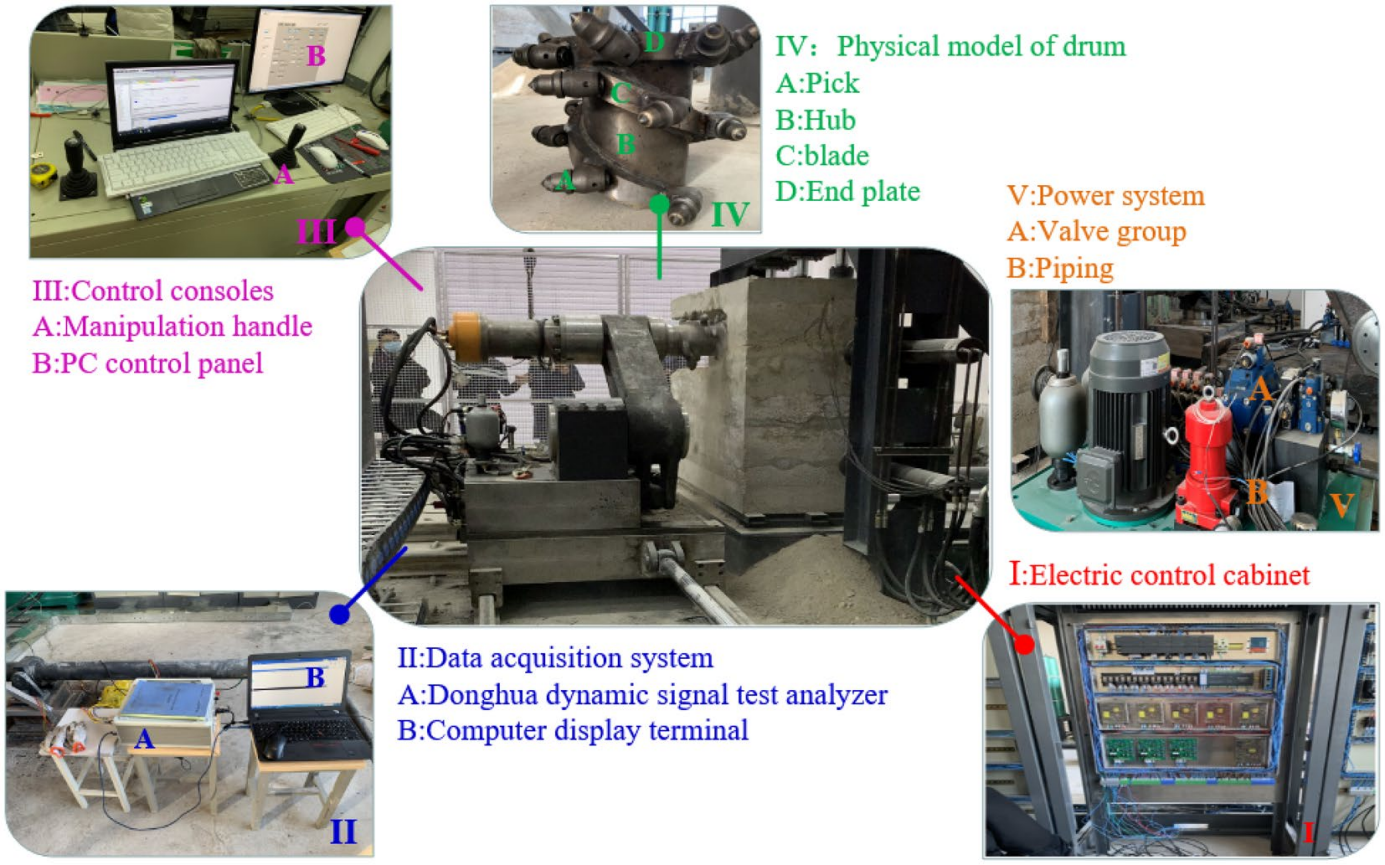
Cutting test bench.

In order to verify the accuracy of virtual simulation results, a test bench for drum cutting was built. Firstly, a physical experimental model of the coal wall was established using materials such as sand, coal powder, and water. And the similarity between the coal wall model and the actual coal wall downhole was proved by uniaxial compression test on the coal wall specimens (Fig. 9). Secondly, a physical model of drum, the power system, the electric control cabinet and the control consoles were built, and the experimental platform was ultimately completed (Fig. 10). Then, according to the simulation work parameters, the drum is controlled by a computer control platform to cut the coal wall, and the load information of the drum during the cutting process under the same working conditions is obtained through a data acquisition system.

**Figure 11:**
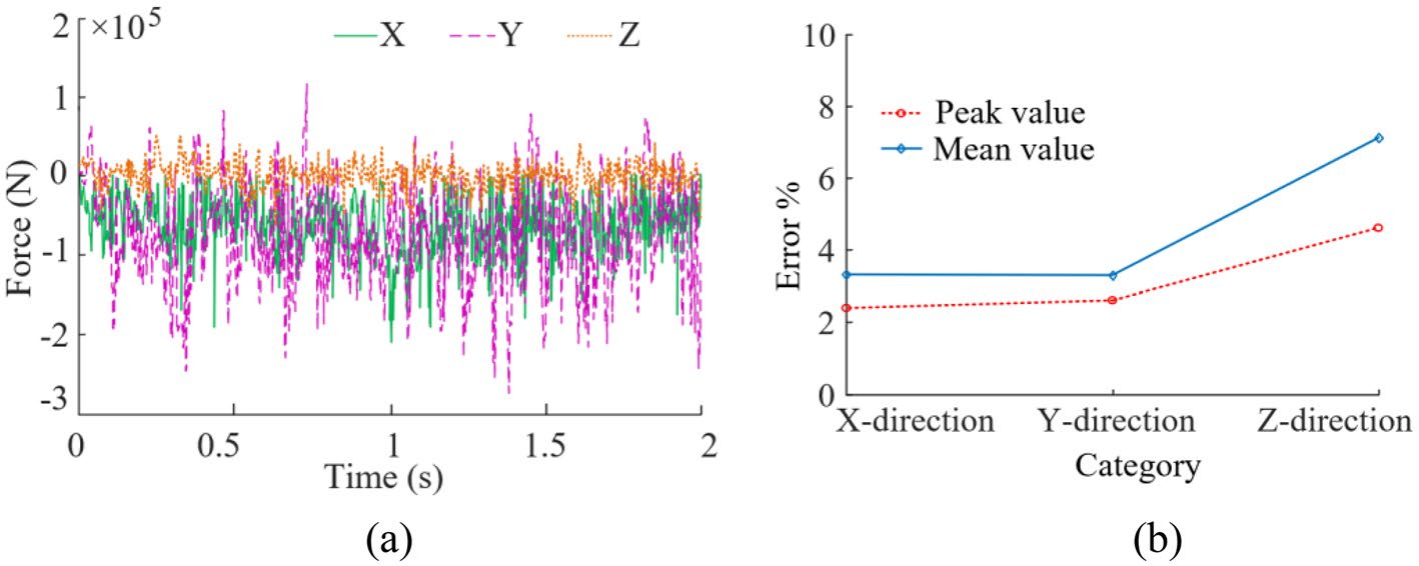
Experimental results. (**a**) Force; (**b**) Errors.

The experimental load of the drum within 2 s is extracted, as shown in Fig. 11a, and the errors between simulation results and experimental results are shown in Fig. 11b.

From Fig. 11, it can be seen that the peak value of the X-direction force is 2.10 × 10^5^ N, and the average value is 3.32 × 10^4^ N. The peak value of the Y-direction force is 2.74 × 10^5^ N, and the average value is 4.14 × 10^4^ N. The peak value of the Z-direction force is 6.26 × 10^4^ N, and the average value is 6.09 × 10^3^ N. The errors between the simulation results and the experimental results are less than 10%. The above indicates that the numerical models established based on the finite element method is effective in studying the dynamic reliability of the drum during cutting coal rock materials.

## Dynamic reliability analysis of drum

### Load characteristics analysis of drum

(1) Influence of rock layer position on drum load

**Figure 12:**
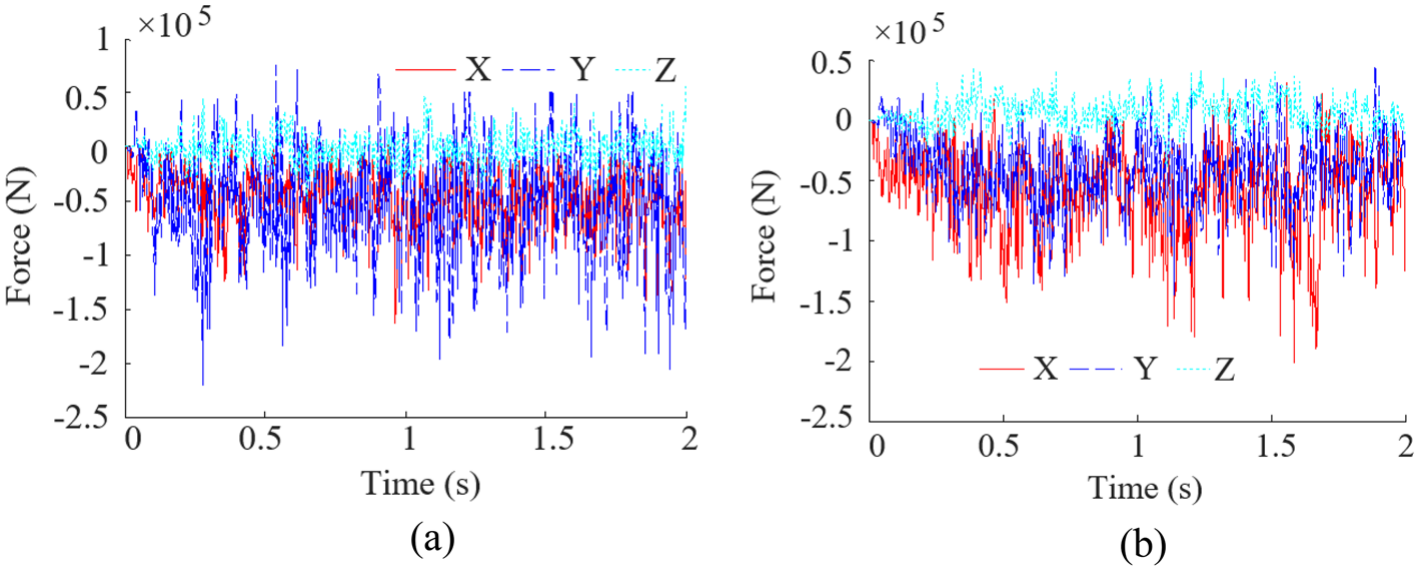
Load curves of the other two working conditions. (**a**) Rock layer is located in position B; (**b**) Rock layer is located in position C.

According to the previous content, there are three typical locations of the rock layer in this coal seam, marked as A, B, C. Through cutting simulation, the drum load curves of the other two working conditions are obtained, as shown in Fig. 12, and the data under the three working conditions are statistically obtained, as listed in Table 3.

**Table 3:** Statistics of drum load under the three working conditions.

Cutting conditions	Mean force (N)		
	X-direction	Y-direction	Z-direction
A	32,144.75	40,127.80	7533.64
B	35,469.23	42,726.85	8309.57
C	39,795.46	36,451.43	5306.79

From Fig. 12 and Table 3, it can be seen that the average value of the three-direction force from high to low is cutting resistance (Y-direction), traction resistance (X-direction), and lateral force (Z-direction). However, in condition C, the traction resistance is the largest, the cutting resistance is the second, and the lateral force is the smallest. This is because the rock layer under working condition C is located at the bottom, and there is a certain amount of bedding when the drum cuts the bottom plate, resulting in greater friction resistance in the traction direction. In the three working conditions, the mean force in Y-direction and Z-direction of working condition B is higher than that in the other two working conditions, and the mean force in X-direction is higher than that in working condition A. This is because when the rock layer is located in position B, the cutting object corresponding to the maximum cutting thickness of cutting is rock material, so the drum are subjected to greater force.

(2) Influence of kinematic parameters on drum load

**Figure 13:**
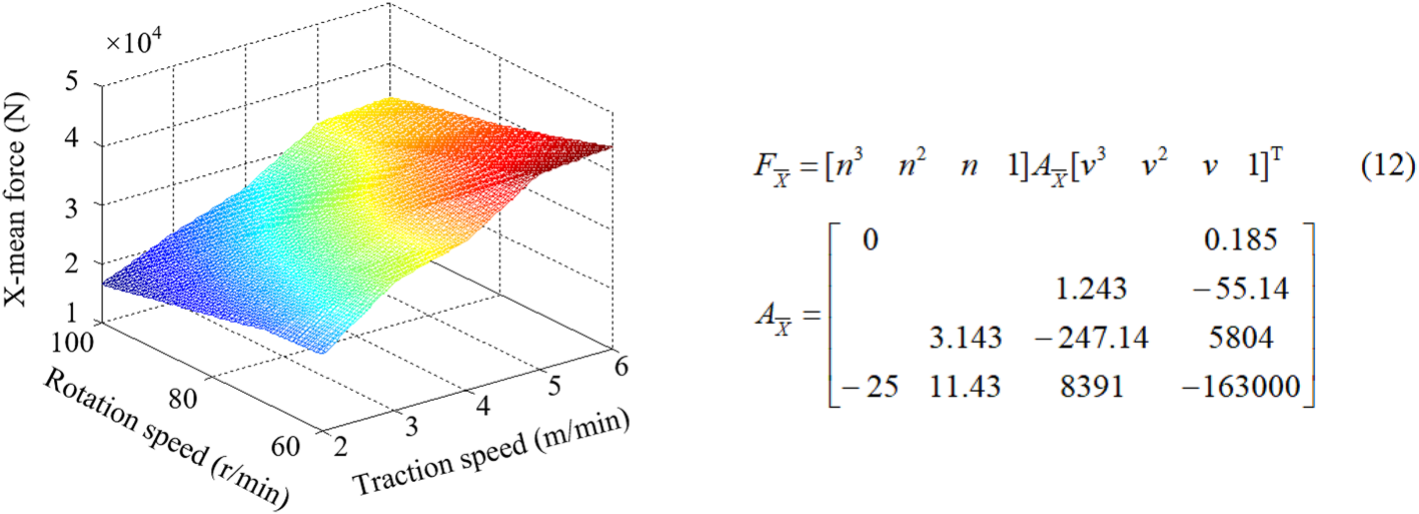
X-mean force.

**Figure 14:**
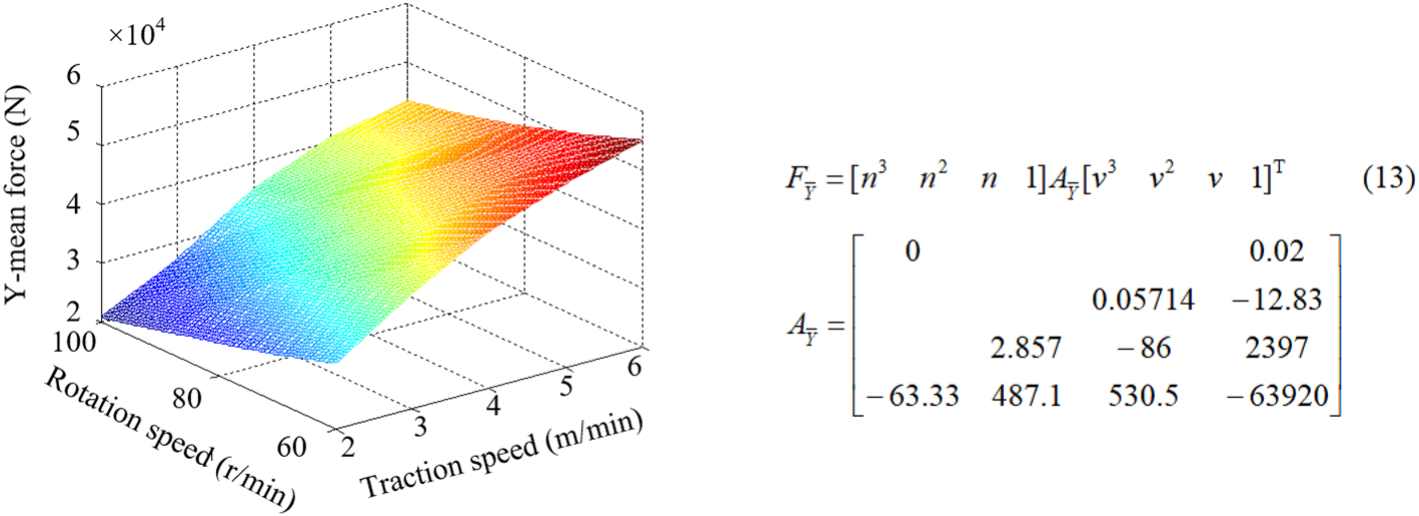
Y-mean force.

**Figure 15:**
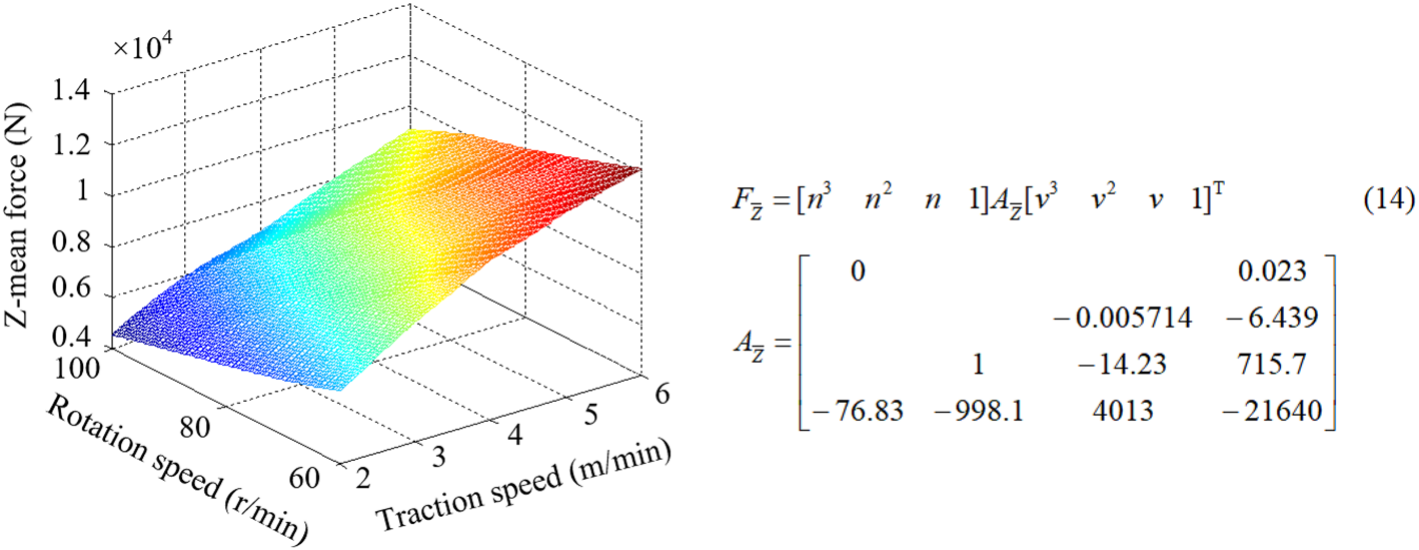
Z-mean force.

Different matching of rotation speed and traction speed can cause the change of cutting thickness of pick, which directly affects the load on the drum and further affects the reliability of shearer. By establishing the numerical simulation models for simulation, the drum load information under different *kinematic* parameters is obtained, as listed in Table 4. The surface fitting is shown in Figs. 13, 14, 15, and the corresponding mathematical models of fitting law are shown in Eqs. (12)–(14).

**Table 4:** Load information of the drum.

Serial number	Rotation speed *n* (r/min)	Traction speed *v* (m/min)	Mean force (N)		
			X-direction	Y-direction	Z-direction
1	60	2	2.30e4	3.13e4	6.21e3
2	70	2	3.18e4	3.98e4	7.56e3
3	80	2	3.73e4	4.61e4	8.92e3
4	90	2	4.10e4	5.15e4	9.82e3
5	100	2	4.41e4	5.51e4	1.05e4
6	60	3	2.23e4	2.91e4	6.15e3
7	70	3	2.95e4	3.69e4	7.92e3
8	80	3	3.37e4	4.21e4	8.27e3
9	90	3	3.76e4	4.70e4	9.35e3
10	100	3	4.20e4	5.05e4	1.04e4
11	60	4	2.05e4	2.66e4	5.44e3
12	70	4	2.71e4	3.39e4	6.77e3
13	80	4	3.21e4	4.01e4	7.53e3
14	90	4	3.63e4	4.34e4	8.39e3
15	100	4	3.83e4	4.55e4	9.13e3
16	60	5	1.85e4	2.42e4	5.00e3
17	70	5	2.43e4	3.04e4	6.29e3
18	80	5	2.99e4	3.74e4	7.03e3
19	90	5	3.30e4	3.91e4	7.98e3
20	100	5	3.62e4	4.10e4	8.36e3
21	60	6	1.66e4	2.19e4	4.78e3
22	70	6	2.28e4	2.76e4	5.76e3
23	80	6	2.67e4	3.15e4	6.82e3
24	90	6	2.90e4	3.50e4	7.45e3
25	100	6	3.42e4	3.70e4	7.95e3

From Figs. 13, 14, 15, it can be seen that when the traction speed is increased from 2 to 6 m/min, the average value of X-direction force corresponding to the rotation speed of 60 r/min, 70 r/min, 80 r/min, 90 r/min and 100 r/min increases by 91.73%, 88.34%, 86.82%, 85.64% and 84.93% respectively, with an average growth rate of 87.49%; The mean value of Y-direction force increases by 76.04%, 73.54%, 71.05%, 69.42%, 68.95% respectively, with an average growth rate of 71.80%; The average Z-direction force increases by 69.08%, 68.29%, 67.83%, 67.20% and 66.32% respectively, with an average growth rate of 67.74%. It can be concluded that the three-direction force increases with the increase of traction speed, but the increase amplitude gradually decreases. The change of traction speed has the largest impact on the traction resistance (X-direction force), followed by the cutting resistance (Y-direction force), and the lateral force (Z-direction force) is the smallest. When the rotating speed is increased from 60 to 100 r/min, the average value of the X-direction force corresponding to the traction speed of 2 m/min, 3 m/min, 4 m/min, 5 m/min and 6 m/min decreases by 27.83%, 28.30%, 28.42%, 29.27% and 30.39% respectively, and the average reduction rate is 28.83%; The average value of Y-direction force decreases by 30.03%, 30.65%, 31.67%, 32.04%, 32.85% respectively, and the average reduction rate is 31.44%; The average Z-direction force decreases by 23.03%, 23.81%, 23.54%, 24.13% and 24.29% respectively, with an average reduction rate of 23.75%. It can be concluded that the three-direction force decreases with the increase of the rotation speed, but the decrease amplitude gradually decreases. The change of the rotation speed has the greatest impact on the cutting resistance (Y-direction force), followed by the traction resistance (X-direction force), and the lateral force (Z-direction force) is the smallest. By comparing the influence of the change of traction speed and rotation speed on the three-direction force of the drum, it is found that the influence of traction speed on the force is more significant.

### Stress reliability analysis of drum

Based on numerical simulation, the stress data of the drum under different kinematic parameters are obtained and listed in Table 5.

**Table 5:** Drum stress statistics.

Traction speed (m/min)	Rotation speed (r/min)					
		60	70	80	90	100
2	Pick body	738.43	704.63	678.49	649.04	621.61
	Pick seat	200.5	324.36	305.15	286.45	263.84
	Blade	82.89	87.18	91.19	95.60	99.77
3	Pick body	791.65	765.48	735.62	690.08	643.4
	Pick seat	364.14	342.38	325.38	316.88	291.72
	Blade	91.75	95.83	98.75	103.46	107.25
4	Pick body	854.51	832.37	812.57	741.81	697.93
	Pick seat	393.72	371.79	352.71	335.41	314.50
	Blade	98.54	103.26	107.86	112.38	116.03
5	Pick body	905.84	874.2	837.58	805.57	753.15
	Pick seat	477.02	425.85	391.85	363.46	340.01
	Blade	105.89	110.69	115.19	119.30	124.15
6	Pick body	951.04	903.87	864.67	821.18	782.34
	Pick seat	558.51	510.00	476.00	442.00	391.54
	Blade	113.11	118.67	121.42	126.65	131.52

According to the analysis in Table 5, when the traction speed is 6 m/min and the drum rotation speed is 60 r/min, the maximum stress on the pick body is 951.04 MPa, and the maximum stress on the pick seat is 558.51 MPa; When the traction speed is 6 m/min and the drum rotation speed is 100 r/min, the maximum blade stress is 131.52 MPa. As the traction speed increases, the stress of the pick body increases to varying degrees. When the traction speed increases from 2 to 6 m/min, the stress of the pick body at drum rotation speeds of 60 r/min, 70 r/min, 80 r/min, 90 r/min, and 100 r/min increases by 28.86%, 28.26%, 27.43%, 26.50%, and 25.92%, respectively, with an average stress increase of 27.394%; As the drum rotation speed increases, the stress on the pick body decreases to varying degrees. When the drum rotation speed increases from 60 to 100 r/min, the stress on the pick body with traction speeds of 2 m/min, 3 m/min, 4 m/min, 5 m/min, and 6 m/min decreases by 15.85%, 18.71%, 18.38%, 16.83%, and 17.77%, respectively, with an average stress reduction of 17.508%. It can be seen that the traction speed has a more significant impact on the stress of the pick body.

In addition, the stress of the pick seat increases with the increase of traction speed, decreases with the increase of drum speed, and increases with the increase of drum speed and traction speed, the stress of the blade increases. This is because the changes in kinematic parameters change the cutting thickness of the pick per unit of time, as well as the quality of coal and rock being cut, thereby changing the forces acting on the pick seat and blade. The higher the traction speed, the greater the cutting thickness of the pick per unit time, and the greater the quality of the crushed coal and rock. After the traction speed increases to a certain value, the amount of coal and rock intercepted by the drum per unit time exceeds the reserved space for coal in the blade, causing blockage in the flow of coal and rock, which cannot be discharged in a timely manner, greatly increasing the load on the blade and making it prone to damage. However, due to the increase in rotation speed, the impact of crushed coal and rock on the blade increases, leading to an increase in stress. Therefore, the reasonable matching of motion parameters is crucial for the reliability of the drum.

### Wear characteristics analysis of drum

According to the coal rock cutting mechanism, during the working process of the drum, the picks are responsible for crushing the coal rock and the spiral blades are responsible for conveying the crushed coal rock to the scraper conveyor. Compared with the hub and end plate, the picks and spiral blades are the main load-bearing parts on the drum that are in direct contact with the coal rock, so the wear of the picks and spiral blades are unavoidable, and they are more prone to failure due to wear, endangering the reliability of the drum and mining safety. In addition, when the rock layer is in the middle position (B), the drum is subjected to the worst load during the cutting process. Therefore, in this study, the wear characteristics of the drum are investigated for this coal seam situation with the picks and spiral blades as the main analysis objects.

To quantitatively analyze the wear situation, the Archard wear model was used to calculate the wear depth:

15 $$V = \sum {\left( {\frac{dV}{{dL}}} \right)} L = \frac{{2N_{t} L}}{{\pi \sigma_{s} }}\cot \theta_{m}$$

where *V* is wear volume, *N*_*t*_ is normal contact positive pressure, *L* is the sliding wear distance, $\sigma_{s}$ is the yield strength of the metal, $\theta_{m}$ is a hard abrasive cone half angle.

Here, a coefficient *K* ($K = \frac{{2\cot \theta_{m} }}{\pi }$) was introduced and substituted into Eq. (15):

16 $$V = \frac{{KN_{t} L}}{{\sigma_{s} }}$$

Thus, the wear depth *H* at a given moment and per unit sliding length is obtained as

17 $$H = \frac{{KN_{t} }}{{\sigma_{s} }}$$

The mathematical model of the total wear depth over a period of time can be expressed as

18 $$H = \int_{0}^{t} {K\frac{{N_{t} (t)}}{{\sigma_{s} }}} dt$$

**Figure 16:**
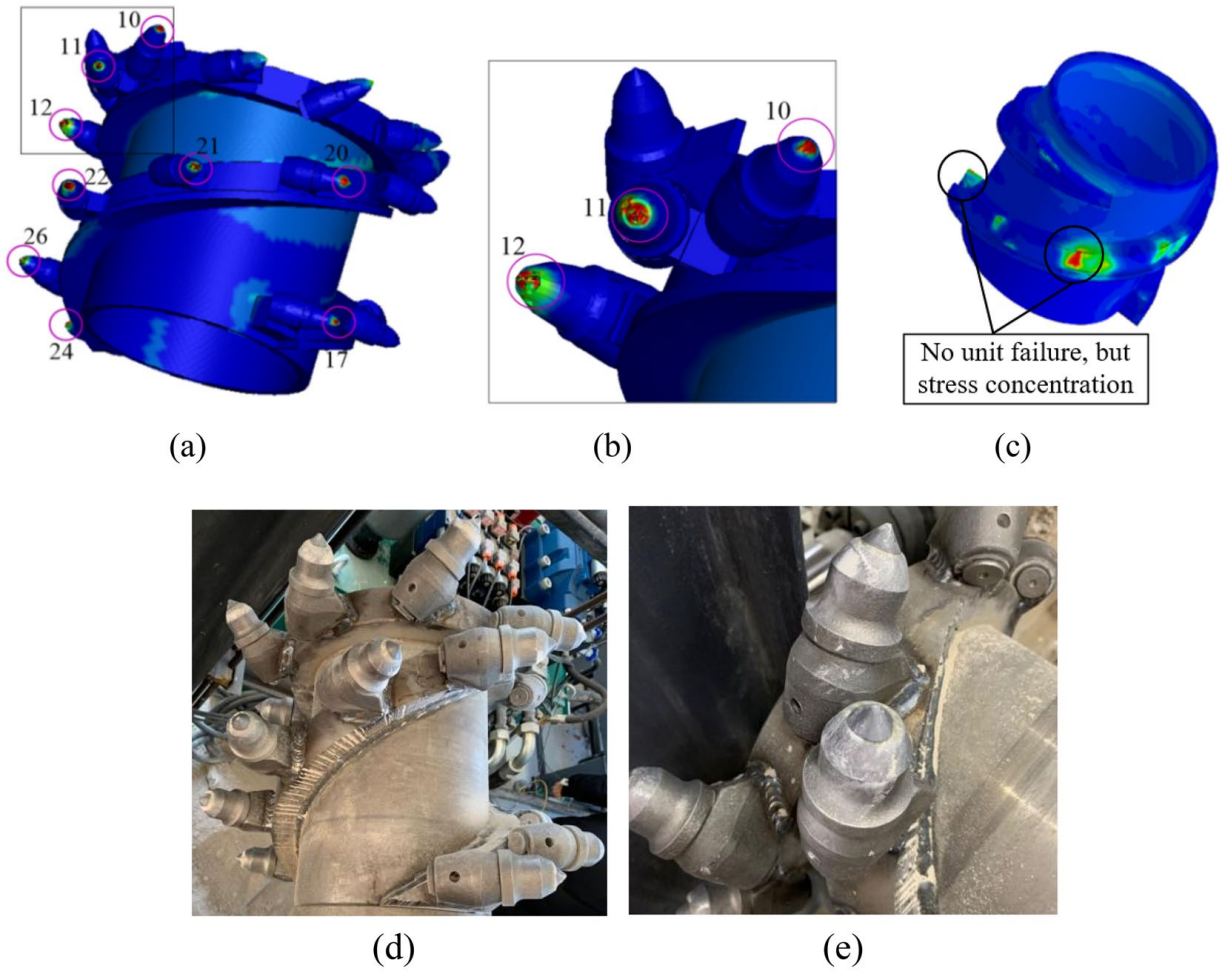
Wear state of the drum. (**a**) Simulation result; (**b**) Wear status of picks in simulation; (**c**) Wear status of spiral blades in simulation; (**d**) Experimental result; (**e**) Wear status picks in experiment.

**Figure 17:**
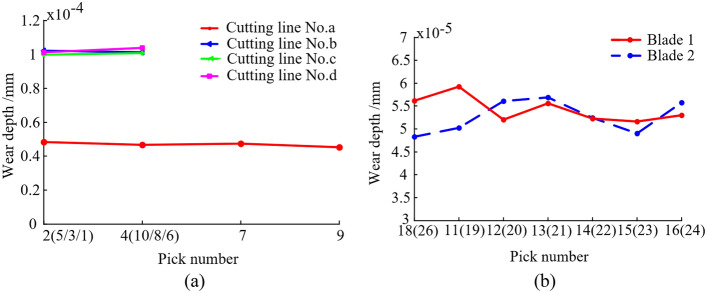
Wear depth of picks. (**a**) Wear depth of the picks on the end plate; (**b**) Wear depth of the picks on the spiral blades.

According to the simulation and experiment test, the wear status of the drum were obtained, as shown in Fig. 16. And the wear depth data of each pick was calculated, as shown in Fig. 17.

The drum is manufactured through processes such as casting and welding, and there is no friction damage on the surfaces of the drum parts before the cutting experiment. It can be seen in Figs. 16 and 17 that the drum undergoes abrasion phenomenon after the cutting, showing obvious groove-like scratches. Each pick on the drum exhibits varying degrees of wear, the picks on the end plate (such as No. 10) and the picks on the spiral blade near the end plate (such as No. 11) are more serious wear. The maximum wear occurs on the No. 10 pick, with a wear depth value of 1.04 × 10^-4^ mm, and the wear of the pick is mainly concentrated in the pick tip and the pick body. However, due to the short cutting time, there is no unit failure of the spiral blade in the simulation model, but there is obvious stress concentration at the outer edge and tail of the blade, so the outer edge and tail of the spiral blade are its weak parts, and wear will occur with the continuation of the cutting time. Therefore, when cutting coal seams with complex conditions, special attention should be paid to the wear status of the picks and spiral blades to avoid dynamic reliability issues of the drum caused by wear failure.

To further investigate the impact of drum wear factors, the mixed orthogonal test method is carried out. Where, the L_6_(3^2^ × 2^1^) mixed orthogonal test table is used to design the test scheme, in which the rotation speed, traction speed and coal loading mode are marked as A, B and C. The coal loading mode is related to the rotational direction of the drum when cutting and conveying coal rock. When the drum cuts and conveys coal rock, if the tip of the picks in the cutting state points to the top of the working face, the conveying mode of the drum on the coal rock is called projectile loading, and when the tip of the picks in the cutting state points to the bottom of the working face, the conveying mode of the drum on the coal rock is called pushing loading. The three levels of rotation speed are 70 r/min (marked as 1), 80 r/min (marked as 2) and 90 r/min (marked as 3) respectively, the three levels of traction speed are 3 m/min (marked as 1), 4 m/min (marked as 2) and 5 m/min (marked as 3) respectively, and the two levels of coal loading mode are projectile loading (marked as 1) and pushing loading (marked as 2) respectively. The finite element models of drum wear under eight different working conditions are set up and simulated according to the test scheme. Furthermore, the wear depth of pick (No. 10) and spiral blade under different working conditions are extracted for analyzed. The results are shown in Table 6.

**Table 6:** Test scheme and results.

Serial number	Factors			Results			
	A	B	C	Pick wear depth/10^−5^ mm		Spiral blade wear depth/10^−6^ mm	
1	1	1	1	10.26		4.28	
2	1	2	2	11.04		5.61	
3	2	3	1	11.07		4.23	
4	2	1	2	9.18		3.55	
5	3	2	1	9.88		2.97	
6	3	3	2	10.57		3.50	

	Pick				Spiral blade		
	A	B	C		A	B	C
*K*_1_	21.30	19.44	31.21	*K*_1_	9.89	7.83	11.48
*K*_2_	20.25	20.92	30.79	*K*_2_	7.78	8.58	12.66
*K*_3_	20.45	21.64	–	*K*_3_	6.47	7.73	–
*k*_1_	10.65	9.72	10.40	*k*_1_	4.95	3.92	3.83
*k*_2_	10.13	10.46	10.26	*k*_2_	3.89	4.29	4.22
*k*_3_	10.23	10.82	–	*k*_3_	3.24	3.87	–
*R*	1.05	2.2	0.42	*R*	3.42	0.85	1.18
*S*	0.46	0.92	0.21	*S*	1.41	0.38	0.59

The analysis of extreme variance can reflect the horizontal volatility of factors. Therefore, according to the value of the extreme variance *R*, the influence order of the factor can be judged. ANOVA can determine the significance of the influence of the factor. The larger the mean square value *S* is, the more significant the influence of the corresponding factor is, and the more important the factor is.

According to the analysis results of pick wear in Table 6, *R*(B) > *R*(A) > *R*(C), which means that the influence order of factors on pick wear is: traction speed, rotation speed, and coal loading mode. In addition, *S*(B) > *S*(A) > *S*(C), which means that the traction speed has the most significant influence on the pick wear, followed by the rotation speed, and the coal loading mode has the least significant influence on the pick wear.

Similarly, according to the analysis results of spiral blade wear in Table 5, *R*(A) > *R*(C) > *R*(B), which means that the influence order of factors on pick wear is: rotation speed, coal loading mode, and traction speed. In addition, *S*(A) > *S*(C) > *S*(B), which means that the rotation speed has the most significant influence on the spiral blade, followed by the coal loading mode, and the traction speed has the least significant influence on the spiral blade.

**Figure 18:**
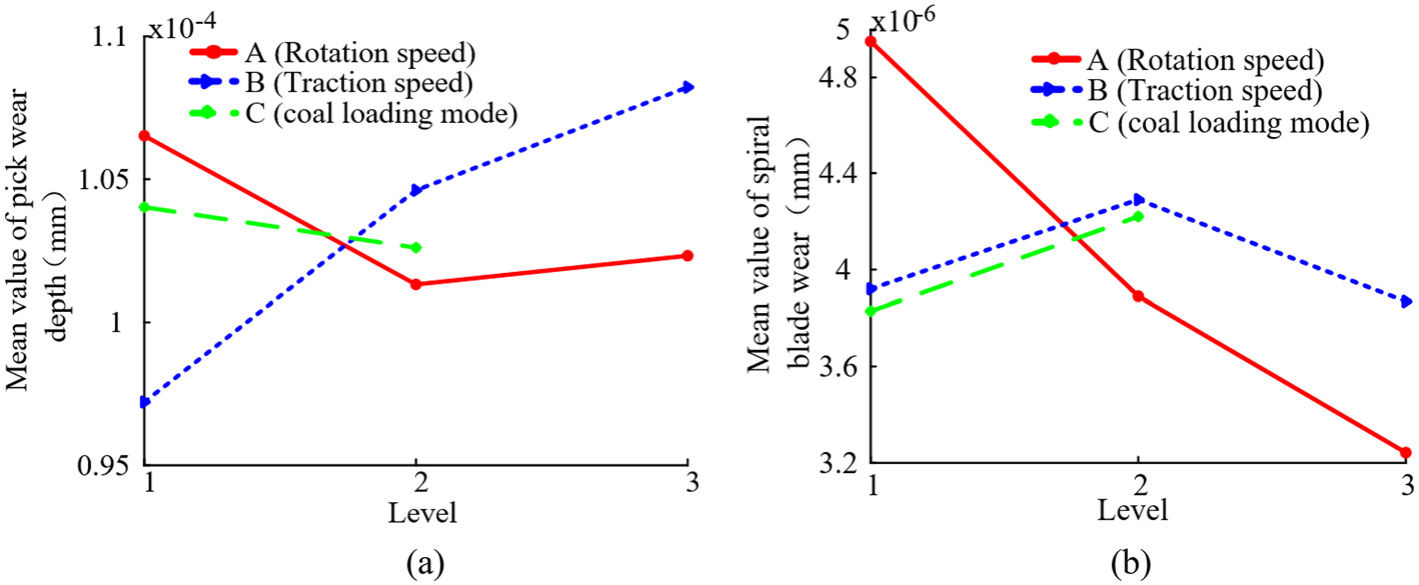
Factor influence trend graphs. (**a**) Factor influence trend of pick wear; (**b**) Factor influence trend of spiral blade wear.

To further analyze the influence law of rotation speed, traction speed and coal loading mode on the wear of pick and spiral blade, the factor influence trend graphs were plotted with the level of each factor as the horizontal coordinate and the mean value *k* as the vertical coordinate, as shown in Fig. 18.

From Fig. 18, it can be seen that with the increase of rotation speed, the pick wear depth decreases and then increases. When the horizontal number is 2, that is, when the rotation speed is 80 r/min, the pick wear depth is the smallest. This is because, by increasing the rotation speed, the cutting thickness of the individual pick decreases in unit time, i.e., the depth of penetration of the individual pick into the material (coal/rock) decreases. Therefore, the cutting resistance of the pick decreases, weakening the extrusion friction between the pick and the coal rock. However, when the rotation speed continues to increase, the sliding friction stroke of the pick increases significantly due to the significant increase of the contact time between the pick and the coal rock in unit time, resulting in an increase in the wear depth. As the traction speed increases, the pick wear depth increases. This is because, increasing the traction speed, the cutting thickness of the individual pick increases in unit time, that is, the depth of penetration of the individual pick into the material (coal/rock) increases. Therefore, the cutting resistance of the pick increases, making the extrusion friction between the pick and the coal rock stronger. In the case of pushing loading, the pick wear depth is close to the wear depth of projectile loading. With the increase of rotation speed, the wear depth of the spiral blade decreases. When the levels is 3, i.e., when the rotation speed is 90 r/min, the wear depth of the spiral blade is the smallest. This is because, by increasing the rotation speed, the cutting thickness of the pick decreases in unit time, so the mass of the crushed coal rock decreases, and the friction between the spiral blades and the crushed coal rock is weakened. When the traction speed level increases from 1 to 2, the wear depth of the spiral blade increases, and the wear depth of the spiral blade decreases when the traction speed level increases from 2 to 3. This is because, when the coal mining machine is hauled at a lower speed, although increasing the traction speed, the lumpiness of the crushed coal rock changes less, but the mass of the crushed coal rock increases significantly in unit time, so the amount of coal rock conveyed by the spiral blades increases, resulting in serious wear of the spiral blades by friction. When the coal mining machine is hauled at a higher speed, although the increase in traction speed will improve the quality of coal rock conveyed by the spiral blade, the increase in the lumpiness of the crushed coal rock makes the contact area between the coal rock and the spiral blade decrease, so the cumulative abrasion depth of the spiral blade by friction decreases. Compared with the projectile loading, the spiral blade wear depth is larger when the drum conveys the coal rock by pushing loading. This is because part of the crushed coal rock will be thrown down directly when projectile loading, so the mass of crushed coal rock to be conveyed by the spiral blade when pushing loading is significantly higher than that of projectile loading, resulting in more serious cumulative wear of the spiral blade.

Based on the above analysis results, it can be concluded that A3B1C1 is the optimal factor level combination that makes the wear of the pick and spiral blade relatively small. That is, the drum rotating speed is 90 r/min, the traction speed is 3 m/min, and the coal loading mode is projectile loading. Of course, in the actual working process of the shearer, the determination of cutting parameters also needs to comprehensively consider other performance, geological conditions and other factors.

## Conclusion

Based on the finite element method, the load characteristics, stress characteristics, and wear characteristics of the drum were studied. The conclusions obtained and the advantages of the method used in this study compared to traditional analysis methods are as follows:

1. With the increase of traction speed, the drum load and stress increases, and with the increase of drum rotation speed, the drum load and the pick stress decreases, while the blade stress increases. The change of traction speed has a more significant impact on the drum load. With the increase of drum rotation speed, the wear of pick decreases first and then increases, and the wear of spiral blade decreases. With the increase of traction speed, the wear of pick increases, and the wear of spiral blade increases first and then decreases. The wear of spiral blades is more serious under the push loading mode.
2. Based on the simulation results to establish the mathematical model of the drum load on each influencing factor, the load of the drum in other different working conditions can be calculated conveniently and quickly, which provides a reference for the design and selection of working parameters of the shearer drum.
3. The wear of the drum is a cumulative and complex process, and it is difficult to observe the wear process of the drum parts by experimental methods. The research on the wear process of the drum can be visualized and controlled through finite element simulation, and the influence of factors on the wear of drum parts can be easily determined.

## Data Availability

The datasets used and/or analyzed during the current study available from the corresponding author on reasonable request.
